# Impact of Thermal Processing on the Composition of *Curcuma longa* Rhizome

**DOI:** 10.3390/foods12163086

**Published:** 2023-08-17

**Authors:** Justyna Zagórska, Wirginia Kukula-Koch, Marcin Czop, Katarzyna Iłowiecka, Wojciech Koch

**Affiliations:** 1 Department of Food and Nutrition, Medical University of Lublin, 4a Chodzki Str., 20-093 Lublin, Poland; justyna.zagorska@umlub.pl (J.Z.); katarzyna.ilowiecka@umlub.pl (K.I.); 2 Department of Pharmacognosy with Medical Plants Garden, Medical University of Lublin, 1 Chodzki Str., 20-093 Lublin, Poland; virginia.kukula@gmail.com; 3 Department of Clinical Genetics, Medical University of Lublin, 11 Radziwiłłowska Str., 20-080 Lublin, Poland; marcin.czop@umlub.pl

**Keywords:** heating, turmeric rhizome, HPLC, curcuminoids

## Abstract

*Curcuma longa* L. (Zingiberaceae), known as turmeric, is a perennial tuberous plant from the genus *Curcuma*, which includes about 100 plant species. The chemical composition of the turmeric rhizome is very diverse. Diarylheptanoid derivatives, also known as curcuminoids (of which curcumin, demethoxycurcumin and bisdemethoxycurcumin are the most important representatives), are the major active constituents of the plant rhizome. Many extracts used in the food and pharmaceutical industries are produced from thermally processed rhizome, when there are significant changes in the composition of the main compounds. Therefore, the aim of the study was to compare how the type of thermal treatment affects the content of curcuminoids and the antioxidant properties of the rhizome. The plant material was subjected to three different methods of thermal processing—microwave heating, boiling and frying in different time intervals. The chemical composition and antioxidant activity of the processed rhizome was evaluated using LC-MS (liquid chromatography–mass spectrometry), HPLC (high-performance liquid chromatography) and spectrophotometric methods (a DPPH test and TPC assay). Obtained results revealed that curcumin was the major curcuminoid present in all samples (113.92 mg/g of the fresh rhizome). Significant correlation between the type and time of the thermal processing and the composition of turmeric samples was revealed. A traditional boiling process lasting for 10 min was the most beneficial process in terms of the curcuminoid content (204 mg/g of curcumin) and antioxidant activity of the samples.

## 1. Introduction

*Curcuma longa* L. (Zingiberaceae), known as turmeric, is a perennial tuberous plant from the genus *Curcuma*, to which about 100 plant species belong [1,2]. The plant has broad leaves and yellow-colored flowers bearing a compound spike with prominent bracts, each subtending a cincinnus of 2–10 flowers, which are attached to each other, forming pouches at the base [3,4,5]. Its natural habitat covers different areas located in subtropical and tropical climates, such as China, India, northern Australia, Malaysia, Thailand and Indonesia [2,4]. In turn, as a crop, it is cultivated in different parts of the world characterized by mild temperatures. The quality of the soil and the environmental conditions in which turmeric is growing are important, as they affect not only its growth, but also the quality (i.e., the composition and content of active compounds) and nutritional value of its tuber, which can translate into its potency and effects on the human body [6,7,8].

The chemical composition of the turmeric rhizome is very diverse. It contains proteins, carbohydrates, fats and also various micronutrients as vitamins (C, B1, B2 and B3) and minerals (sodium, iron, calcium and potassium). Its tubers are rich sources of terpenes and phenolic components [9,10,11,12]. Among the latter compounds present in this plant, diarylheptanoids, also known as curcuminoids (of which curcumin, demethoxycurcumin and bisdemethoxycurcumin are the most important representatives), diarylpentanoids (e.g., 1,5-bis(4-hydroxy-3-methoxyphenyl)-penta-(1E,4E)-1, 4-dien-3-one) and others (vanillin, vanillic acid, (Z) ferulic acid, (E) ferulic acid, (E)-4-(4-hydroxy-3-methoxyphenyl)but-3-en-2-one), were distinguished. Volatiles present in the extracts from turmeric rhizomes are abundantly represented by different groups of terpenes, like monoterpene hydrocarbons, sesquiterpenes or diterpenes. In addition, there are steroids in the turmeric rhizome: stigmasterol, gitoxigenin, β-sitosterol and 20-oxopregn-16-en-12-yl acetate [10].

Due to a marked concentration of active components, *Curcuma longa* exhibits a broad spectrum of medicinal effects. Therefore, it has been used for more than 2500 years in traditional Islamic, Pakistani, Chinese and Ayurvedic medicine centers [13,14,15,16]. In folk medicine, turmeric was administered to treat the disorders of the upper parts of the respiratory tract, in jaundice and to relieve symptoms of a cold [17]. Also, the rhizome showed antiseptic, anthelmintic, anti-inflammatory, blood-circulation-enhancing, anticoagulant, antirheumatic and antimalarial properties. Moreover, it was topically administered to help heal cuts and wounds, and to treat pimples. *Curcuma longa* was used to combat bronchitis, kidney infections, diabetes, obesity and indigestion. Traditional medicine in various regions of the world has used various preparations of the turmeric rhizome, such as juice, tonic, decoction, powder or paste [13,18,19,20,21,22]. Curcumin—the major curcuminoid present in the turmeric rhizome—possesses a very wide spectrum of health-promoting properties, thanks to which it can be used as a substance supporting homeostasis in the body (prophylactic effect) with its proved anti-inflammatory, anti-cancer, antioxidant and anti-diabetic properties, and cardiotonic action [17,23,24,25]. For centuries, turmeric has been widely used, primarily in the cuisines of Asian countries, mainly China and India, as a food colorant, as an ingredient used in dietary supplements, as a spice that imparts a distinctive flavor to dishes and as a food preservative [2,26,27].

Supplements containing various extracts and preparations of the turmeric rhizome or single curcumin are mostly used as a supporting agent in arthritis, obesity and liver diseases [28,29,30,31,32]. However, the composition of such products is not being standardized and various preparations of the turmeric rhizome can be used. Many extracts are produced from a thermally processed rhizome, when there are significant changes in the composition of the main compounds. Therefore, changes in the composition of curcuminoids that occur during thermal processing are important during the production of turmeric products, as well as from the nutritional point of view, because this raw material is one of the most commonly used spices.

The biological activity of the *Curcuma longa* L. rhizome was also analyzed after thermal processing. The most commonly used thermal methods were boiling, frying and microwaves. It has been proven that these processes change the composition and properties of the turmeric rhizome extracts. Most of the research conducted in this field did not show unequivocal results. Apart from the type of the thermal method used, the processing time was also an important factor [33,34,35,36,37]. Performed studies revealed that thermal treatment did not always result in a reduction in or loss of the beneficial properties of a plant material. It was shown that the thermal treatment of curcumin causes its degradation and formation of new compounds. It was noticed that the products of the thermal transformation of curcumin may be characterized by increased bioavailability and absorption by cells than the initial compound and show increased effects, e.g., antioxidant or anti-cancer. Both of these factors could translate into a stronger health-promoting potential of thermally processed curcumin and its breakdown products [35,38,39].

Considering the changes in the plant’s fingerprint under the influence of temperature, the authors found it crucial to study the impact of everyday cooking routines on the composition and antioxidant activity of the turmeric rhizome. The aim of the study was to compare how the type of thermal treatment affects the content of the main active compounds of the turmeric rhizome—curcuminoids—and the antioxidant properties of the rhizome. To achieve the goal, the plant material was subjected to three different methods of thermal processing—microwave heating, boiling and frying in different time intervals. Thermal processes were adjusted in a way to mimic thermal treatment used in traditional home practice, during the preparation of meals. The results obtained during the research allow for assessing how the culinary processes and their duration may affect the content of curcuminoids in the turmeric rhizome and its antioxidant potential. There is some data on the effect of heat treatment on changes in the composition of turmeric rhizomes; however, the presented study is a completely new approach showing how typical, simple thermal methods used every day during food preparation affect changes in the chemical composition and antioxidant activity of the turmeric rhizome.

## 2. Materials and Methods

### 2.1. Plant Material and Thermal Treatment

Investigated material: Fresh turmeric rhizomes (*Curcuma longa* L.) were purchased in Lublin, Poland in a local market. The rhizomes were cut into slices with the weight of 2 g and directly used for further analyses or thermal processing.

Thermal processing: Portions of 2 g of fresh turmeric rhizomes were subjected to several kinds of thermal processing with variable times of heating. The plant material was cut into slices and then subjected to three different thermal processes: boiled in hot deionized water (100 °C), fried on a ceramic pan with no addition of oil or processed in a microwave oven (Zelmer, Rzeszów, Poland) using a microwave power of 800 W. In all types of heat treatment, the samples were processed for 1, 2, 5 and 10 min. All processes were repeated 3 times. Microwave treatment for 10 min charred the rhizome; therefore, these samples were not included in further studies.

### 2.2. Chemicals and Reagents

Methanol (Avantor Chemicals, Gliwice, Poland) was used to obtain the extracts from the turmeric rhizome. For a chromatographic analysis, ultrapure water (a resistivity of 18.2 MΩ*cm) and acetonitrile of an HPLC grade (JT Baker, Phillipsburg, NJ, USA) were applied as solvents, constituting the mobile phase. The antioxidant activity assays were performed using sodium carbonate of a reagent grade (Avantor Chemicals, Gliwice, Poland), a Folin–Ciocalteau reagent (StanLab, Lublin, Poland) and 2,2-diphenyl-1-picrylhydrazyl (DPPH) (Sigma Aldrich, St. Louis, MO, USA). The standard reagents used in the study were gallic acid, curcumin, demethoxycurcumin and bisdemethoxycurcumin (Sigma Aldrich, St. Louis, MO, USA). High-purity deionized water (a resistivity of 18.2 MWcm), obtained using an Ultrapure Millipore Direct-Q-R 3UV (Millipore, Bedford, MA, USA), was used throughout the analysis.

### 2.3. Extraction

Each turmeric sample after a given heat treatment of a certain duration was poured with 10 mL of methanol and extracted on an ultrasonic bath for 10 min. The sample was then transferred to 10 mL glass tubes and centrifuged twice. Later, the supernatant was filtered through the tissue paper filter type 388 (Chemland, Stargard, Poland) into glass dishes. In the next step, the solution was transferred to a 100 mL round-bottom flask, which was connected to a vacuum evaporator to evaporate the excess solvent under reduced pressure (<100 hPa) at a temperature not exceeding 40 °C. To prepare the samples for analyses, 5 mg of the dried residue was weighed and dissolved in 2 mL of methanol.

### 2.4. LC-MS Analysis of the Obtained Extracts

For qualitative purposes, the samples were analyzed for their fingerprint and composition using the HPLC-ESI-QTOF-MS/MS (high-performance-liquid-chromatography–electrospray-ionization–quadrupole-time-of-flight–tandem-mass-spectrometry) method. Detailed information is presented in the Supplementary Material.

### 2.5. The Conditions of Qualitative and Quantitative HPLC Analyses

A portion was taken from each previously prepared extract and filtered into 2 mL vials through a nylon syringe filter with a pore diameter of 0.45 μm. The prepared solutions were analyzed using an HPLC chromatograph.

The chromatographic analysis was performed using a Prominence-i LC-2030 3D HPLC system equipped in a PDA detector (Shimadzu, Kyoto, Japan), a degasser, an autosampler and a quaternary pump. The separation of the extracts’ constituents was achieved on a ReproSil-Dur Basic-C18 column (250 × 4.6 mm, dp *=* 5.0 μm) manufactured by Dr. Maisch (Ammerbuch-Entringen, Germany). The settings of the chromatograph were as follows: a thermostat temperature of 25.0 °C, flow rate of 1 mL/min, injection volume of 10 µL, UV detection range of 190–500 nm and DAD detection wavelengths of 280 and 320 nm. A single analysis lasted 25 min and the mobile phase composition (solvent A—ultrapure water/solvent B—acetonitrile) was as follows: 0 min—40%:60%, 5 min—30%:70%, 15 min—20%:80%, 16 min—5%:95% and 19 min—40%:60%. Curcumin, demethoxycurcumin and bisdemethoxycurcumin standard solutions (1.9 mg/mL in methanol) were used for quantitative determinations. The calibration curves were plotted for the three standards. The following equations were obtained for curcumin: y *=* 734,873x + 17,707 (R^2^ *=* 1.0000), for demethoxycurcumin: y *=* 766,701x + 22,410 (R^2^ *=* 0.9994) and for bisdemethoxycurcumin: y *=* 766,174x − 7491.1 (R^2^ *=* 0.999). The linearity range covered the determined concentrations of curcuminoids in the samples and was calculated as 10–300 mg/g of the fresh rhizome. The recovery of curcuminoids was equal to 95, 92 and 91% for curcumin, demethoxycurcumin and bisdemethoxycurcumin, respectively. The repeatability of the quantitative determinations was higher than 95% for every compound. The limit of detection (LOD) values expressed as signal-to-noise (S/N) times 3 for the compounds in the prepared method were calculated to be 0.212, 0.226 and 0.232 mg/g (*n* = 5), whereas the limit of quantification (LOQ) calculated as S/N times 10 was 0.636, 0.678 and 0.696 mg/g (*n* = 5) for curcumin, demethoxycurcumin and bisdemethoxycurcumin, respectively.

### 2.6. The Determination of the Antioxidant Potential of the Obtained Extracts with Spectrophotometric Methods

#### 2.6.1. Determination of Total Phenolic Content (TPC)

The total content of polyphenolic compounds in the fresh and thermally treated turmeric rhizome was determined using a Thermo Fisher Scientific Evolution 300 UV-Vis spectrophotometer (Thermo, Waltham, MA, USA) using the Folin–Ciocalteu reagent method as previously described [40].

Initially, 0.5 mL of the studied extract was transferred into a 50 mL volumetric flask. Then, 30 mL of purified water and 2.5 mL of the Folin–Ciocalteu reagent were added. The solution was mixed and allowed to stand for 1 min. Then, in no more than 8 min, 7.5 mL of a 20% Na_2_CO_3_ solution was added and the whole was made up to 50 mL with distilled water. The obtained mixture was set aside for 2 h in a place with limited access to light. The mixture was stirred again and used for a spectrophotometric determination in 1 cm cuvettes, which were placed in an apparatus. The absorbance was measured at 760 nm.

The calibration curve equation was calculated based on the measurement of gallic acid absorbance. The standard solutions were prepared in a similar manner to the studied samples. The concentrations of the standard solutions were 50, 100, 200, 300 and 500 mg/L. Using the prepared calibration curve, the total content of polyphenolic compounds in the studied samples was calculated in gallic acid equivalents.

#### 2.6.2. Free-Radical Scavenging Activity (DPPH Test)

The antioxidant capacity assay was performed using the DPPH radical (1,1-diphenyl-2-picrylhydrazyl) scavenging activity test, according to a previously described protocol with appropriate modifications [40]. Briefly, 25 μL of each extract was mixed with 1775 μL of the previously prepared radical solution in methanol (absorbance: 0.9). The mixture was then left for 30 min in a place protected from light. After the designated time, the solutions were transferred to a 1 cm cuvette and the absorbance at 515 nm was measured using a UV-Vis Thermo Fisher Scientific Evolution 300 spectrophotometer. Using the formula below, the antioxidant potential was calculated and expressed as % inhibition.

$$I\left(inhibition\right)\left[\%\right]=\left[\frac{A_{0}-A_{t}}{A_{0}}\right]*100\%,$$

where

A_0_—the initial absorbance of DPPH;

At—the absorbance of the studied solution at the end of the reaction.

### 2.7. Statistical Analysis

A statistical analysis was performed using Statistica 13.3 (StatSoft, Kraków, Poland). Data are presented as the mean with SD. The Shapiro–Wilk test was used to evaluate the normality of the data distribution. A two-way Anova (with one qualitative factor and repeated measures) with the Bonferroni post hoc test were used. Results were considered statistically significant when *p* ≤ 0.05.

## 3. Results and Discussion

### Qualitative and Quantitative Analysis of the Thermally Processed Turmeric Rhizome Using LC-MS and HPLC Methods

First, the fingerprint of the turmeric extract was obtained in the HPLC-MS analysis (see Figure S1 in the Supplementary File). The mass chromatogram revealing the constituents of the turmeric extract was recorded in the negative ionization mode. Among the detected *m*/*z* signals, three curcuminoids found their place and were eluted from the column after 25.5 min. The recorded spectra of curcumin, demethoxycurcumin and bisdemethoxycurcumin are shown in Figure S2 in the Supplementary File.

**Figure 1:**
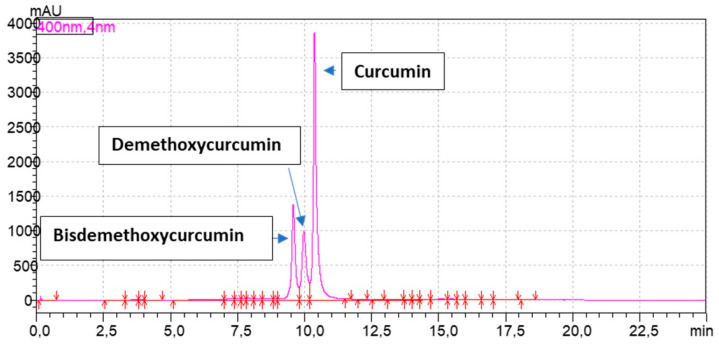
Chromatogram of the fresh extract from *Curcuma longa*.

The elaborated chromatographic methods enabled the separation and qualitative and quantitative determination of three major curcuminoids in the investigated plant material—curcumin, demethoxycurcumin and bisdemethoxycurcumin. Figure 1 presents a sample chromatogram of the extract from the fresh rhizome with identified major compounds visible in 400 nm in the HPLC instrument. Further chromatograms of the analyzed extracts from a processed plant are presented in the Supplementary File (Figure S3).

Based on the performed chromatographic determinations, the concentration of the studied curcuminoids was calculated in each extract obtained from a thermally processed turmeric rhizome. Obtained results were expressed in mg per 1 g of the plant material and are presented in Table 1. The performed analysis revealed that curcumin was the major curcuminoid in all studied samples (fresh and processed ones), which is in agreement with the majority of studies, suggesting curcumin as a major curcuminoid in the turmeric rhizome [29,41,42]. However, there are also studies indicating that, depending on the origin, the composition of active components in the rhizome of *Curcuma longa* may vary. Charoenchai et al. [33] revealed bisdemethoxycurcumin (4.57–5.76% *w*/*w*) to be the major curcuminoid in the turmeric rhizome, followed by curcumin (2.69–3.77% *w*/*w*) and demethoxycurcumin (1.84–2.52% *w*/*w*). Obtained results show that the type of culinary process significantly affects the content of active compounds. Appropriate chromatograms are presented in the Supplementary Material (Figure S3). The type of the cooking process has the greatest impact on the content of the investigated compounds in the extracts obtained from the processed turmeric rhizome, which is more significant than the time of the particular processes. Between the results obtained for particular types of treatment, there is a high positive Pearson correlation coefficient for all the compounds, which means that the content of curcuminoids increases with the duration of the process (Table 2). This would probably be explained with the intensification of the degree of extraction of the active compounds as a result of thermal damage to the cells of the studied plant material, and thus an easier and more effective extraction of curcuminoids. However, the opposite trend was observed for microwave processing. For this, the correlation coefficient was negative, so the longer the duration of the process, the lower the content of curcuminoids. This is probably related to the negative impact of microwaves on the studied compounds, and thus their lower content in the analyzed extracts. In the case of the frying process, for bisdemethoxy- and demethoxycurcumin, Pearson correlation coefficients have a positive value, which means an increase in the content of these compounds along with the lengthening of the cooking time. On the other hand, a negative value is assumed for curcumin, the content of which decreases with the increasing time of subjecting the raw material to frying.

A Pearson coefficient value in the range of 0.5–1.0 indicates a strong correlation. The results presented in the table showed that each of the preformed culinary processes significantly affected the content of curcuminoids in the turmeric rhizome. However, the best results, regarding the content of curcuminoids, were obtained during boiling and frying, depending on particular compounds. For curcumin, short-time frying significantly elevated the amount of the compound in the rhizome. In the case of bisdemethoxycurcumin, long boiling up to 10 min increased the concentration of the compound by almost double. The highest total content of curcuminoids was determined after frying lasting for 10 min (346.9 mg/g), followed by boiling for the same time (342.9 mg/g), which suggests that both processes increased the content of the determined compounds in the extract almost equally. The least favorable process, regarding the content of the active compounds, was thermal heating using microwaves. After 5 min, the content of identified compounds was the lowest—the concentration of particular curcuminoids was reduced 3–4 times—which proves a very detrimental influence of microwave processing on the content of curcuminoids in the turmeric rhizome.

The antioxidant activity of curcumin and other derivatives results from their chemical structure—the presence of phenolic groups in its structure, which are electron-donating groups—which has been confirmed with various in vitro and in vivo tests [43]. However, the influence of different thermal processes on the antioxidant activity of the turmeric rhizome is not fully understood and obtained results are inconclusive. Therefore, results of the present research may shed a new light on this subject.

The antioxidant potential of the studied samples was determined using the DPPH radical method, while the total content of polyphenolic compounds was assessed using the Folin–Ciocalteu method (Table 3). The obtained results were strictly correlated, which was expected considering that the TPC method is often used to evaluate the reducing power of a plant extract and is considered as one of the most reproducible techniques for assessing the antioxidant activity of plant-derived samples [44]. The highest percentage of free radicals’ scavenging was determined for turmeric rhizomes boiling for 10 min (51.6%, which was equal to 0.055 µM of a neutralized DPPH radical). For the same parameters, one of the highest TPC results (527 mg/L) was also measured. During the cooking process, the same relationship was observed as when studying the concentration of particular curcuminoids. As the duration of treatment was increased, the antioxidant potential and TPC value were also increased. Similar results were obtained by other authors [37], proving that the increase in both the duration and temperature of thermal treatment significantly increased the content of polyphenolic compounds. Similar results were obtained in the case of frying. Extending the duration of the process had a positive effect on the antioxidant potential and the content of polyphenolic compounds. It seems that the antioxidant activity of the samples was strictly correlated with the concentration of curcumin, which was proved to be the major curcuminoid and this compound was mainly responsible for the antioxidant activity of turmeric rhizomes. In samples boiled and fried for 10 min, the concentration of curcumin was very high in comparison to the fresh rhizome, which was 204 and 224 mg/g for samples boiled and fried for 10 min, respectively. However, the high antioxidant activity of the turmeric rhizome was also connected to the concentration of other curcuminoids and their total effect seemed to be synergistic. It was visible, considering the antioxidant activity of the samples boiled and fried for 10 min, in which the concentration of bisdemethoxycurcumin and demethoxycurcumin was very high. On the other hand, in samples treated for 5 min using a microwave, for which the determined antioxidant activity was the lowest, the concentration of curcumin was high, but the content of two other curcuminoids was the lowest, which might prove that all three curcuminoids are significant for the high antioxidant activity of the turmeric rhizome. Studies by Dahmke et al. [35] also proved that curcumin previously subjected to the cooking process (frying on a pan at 250 °C) showed higher bioactivity—both antioxidant and anti-cancer properties. A reverse relationship was observed in the case of microwave processing. Extending the time of subjecting the raw material to microwave heating had a negative impact on the determined parameters. The lowest TPC values (327 mg/L) and the lowest antioxidant activity expressed as a percentage of inhibition (19.7%, which was equal to 0.021 µM of a neutralized DPPH radical) were determined for this type of thermal treatment during 5 min.

Plants from the Zingiberaceae family are highly valued for their properties and taste, and they are commonly used as spices and other food additives; therefore, many studies on the influence of thermal processing on the composition and activity of these products have been performed so far. However, the obtained results are very often inconclusive, both in terms of the observed changes in composition and activity [45,46]. Prathapan et al. [37] investigated the effect of time (10–60 min) and temperature (60–100 °C) in the thermal treatment of a fresh turmeric rhizome on the content of curcuminoids, phenols (TPC) and the activity of polyphenol oxidase (PPO), i.e., an oxidizing enzyme responsible for the browning of plant tissues. It was noticed that the processed samples were characterized by a higher total phenolic content from the unprocessed samples. The amount of these compounds was increasing gradually together with the elevation of temperature from 60 to 80 °C. The TPC value did not show any changes in the temperature range between 90 and 100 °C. Interestingly, there was no significant change in curcuminoid content among the heat-treated samples. In contrast, samples that were dried in the sun had a significant reduction in curcuminoid concentrations. The effect of heating on PPO was also proven in this study. As a result of heating, the activity of this enzyme decreased, and after treatment lasting for 30 min at 80 °C, PPO was almost totally inactivated.

In other studies, the impact of the thermal processing of fresh turmeric tubers (the Prathiba variety) was assessed to determine the changes in single components’ concentration after boiling (40, 60 and 90 min) in water and then drying in the sun (a temperature max. of 37 °C). An increasing processing time went together with the decomposition of curcumin, essential oils, oleoresin and starch. After the shortest treatment of 40 min, the content of curcumin was 5.91%, that of essential oils was 3.60%, that of oleoresin was 13.33% and that of starch was 66.96% in the tested samples. However, under a longer treatment (90 min), the values were decreased to 5.12%, 3.33%, 13.08% and 62.44%, respectively [36]. Cortez et al. compared the effects of traditional cooking and pressure cooking on the content of phenolic compounds, curcumin and various types of fatty acids in turmeric. Both boiling and cooking combined with high pressure resulted in a loss of phenolic compounds in the raw material. On the other hand, both these methods resulted in an increase in the content of polyunsaturated acids from 18% for the control sample to 33% after boiling and 48% after pressure cooking. It was concluded that pressure cooking is a better thermal treatment method for turmeric than traditional cooking, as it provides a higher content of monounsaturated and polyunsaturated acids, but a lower amount of saturated fatty acids [34].

Charoenchai et al. [33] analyzed the compositional changes in *Curcuma longa* after thermal processing. The studied material, fresh rhizome, was divided into two groups. The first was subjected to cooking for 30 min at 80 °C, while the second part was left untreated. The cooked and uncooked samples were then subjected to three methods of drying, respectively: in a microwave oven for 5 min at 450 W, in a microwave oven for 5 min at 850 W and in an oven for 5 h at 60 °C. In this study, the effect of the applied heat treatment on, among others, the total content of curcuminoids, the concentration of individual curcuminoids (curcumin, demethoxycurcumin and bisdemethoxycurcumin) and the content of essential oil and an ethanol-soluble extract was evaluated. The obtained results for the previously mentioned components of turmeric showed that there were no significant differences in oven-dried or microwave-dried samples. The total content of curcuminoids was similar in all samples and ranged from 7.85 to 8.58% *w*/*w*.

There are also other studies on the thermal processing of the ginger rhizome, another commonly used spice and food ingredient. One of the studies also evaluated the effect of culinary processing and its duration on the antioxidant activity of the ginger rhizome (*Zingiber officinale* L.) belonging to the same family—Zingiberaceae. The described results significantly differed from those of the present study. In the case of ginger, an increase in the antioxidant properties was observed after microwave treatment. The highest decrease in the antioxidant properties occurred after frying. Simple boiling in water also resulted in a decrease in the antioxidant activity; however, these changes were much lower in comparison to frying [47].

The scientific literature shows many examples of a differentiated antioxidant potential and composition of turmeric tubers. The results obtained in our studies deliver more scientific data on this topic. As turmeric is often heated prior to consumption, the knowledge on the behavior of its single components is needed. Our studies showed a similar tendency to the results published by other researchers, who showed that the longer the time of the thermal treatment (cooking), the content of polyphenolic compounds in a sample and its antioxidant activity increased [35,37].

However, our results are not fully in agreement with the results of other authors, who did not reveal a significant effect of thermal treatment on the content of curcuminoids [33,37]. The published results of analyses by other researchers indicated a decrease in curcumin content in *Curcuma longa* under the influence of cooking, which is inconsistent with the results obtained in the present study [34,36]. Results of the present research provide new analytical data and show that thermal treatment significantly affects the total content of polyphenolic compounds and the concentration of individual curcuminoids, which, in total, translates into changes in the antioxidant potential of particular extracts. Moreover, both the type and time of the thermal processing influence the composition and biological activity of the samples. It was proved that for classical boiling in water and frying, the longer processing of turmeric is more beneficial. Interestingly, in the case of thermal treatment using microwave radiation, the situation was reversed. Therefore, it seems that this type of thermal processing causes the most negative changes in the composition and antioxidant activity of the turmeric rhizome.

## 4. Conclusions

The results of the present study revealed that curcumin was the major curcuminoid present in all samples. Significant correlation between the type and time of the thermal processing and the composition of turmeric samples was revealed. The mildest method of thermal processing, in terms of the impact on the concentration of active phenolic compounds (curcuminoids) and antioxidant activity, was the traditional boiling process lasting 10 min. In contrast, the lowest concentration of curcuminoids and antioxidant activity were calculated for the samples heated with microwave energy for 5 min. Moreover, it was proved that in the case of boiling and frying, the antioxidant activity of the samples was increasing with the elevation of the processing time. In turn, the extension of the microwave treatment time resulted in a significant decrease in the antioxidant activity of the samples. The conducted research showed that the most beneficial type of thermal treatment of the turmeric rhizome was traditional cooking using boiling water. Based on the obtained data, it can be concluded that microwave radiation has the least favorable effect on the content of active components and antioxidant activity of the turmeric rhizome. The obtained results may be of practical importance and provide valuable tips for people preparing dishes at home, as well as on an industrial scale, where the turmeric rhizome is used as a valuable food ingredient. The results showed that cooking had the most beneficial effect on the content of curcuminoids, as well as the antioxidant activity of the rhizome, while microwave processing seemed to be the least favorable process for the thermal treatment of this raw material.

The present study also has some limitations. The scientific goal of the experiment was focused only on the quantitative changes in the composition of the turmeric rhizome, without searching for new derivatives, which may be synthetized during thermal processing. Therefore, changes in the biological activity of the turmeric rhizome, due to thermal processes, may result not only from quantitative but also qualitative changes in the composition of the plant material, but more studies are needed to fully elucidate this process.

## Figures and Tables

**Table 1:** Influence of various thermal processes on the content of three curcuminoids in the rhizome of *Curcuma longa*.

	Boiling (*n* = 3)	Frying (*n* = 3)	Microwave (*n* = 3)
	Curcumin (mg/g)		
**Fresh**	113.92 ± 12.24 ^a^	113.92 ± 12.25 ^c^	113.92 ± 12.25 ^b^
**1 min**	125.33 ± 9.61 ^ab^*^^^	246.00 ± 12.77 ^b^*	200.67 ± 18.88 ^c^^
**2 min**	102.93 ± 8.30 ^a^*	203.00 ± 23.43 ^ab^*^#^	73.50 ± 9.56 ^ab#^
**5 min**	164.67 ± 17.50 ^bc^^	174.67 ± 22.37 ^a#^	35.97 ± 2.07 ^a^#^
**10 min**	204.00 ± 21.38 ^c^	224.00 ± 26.96 ^ab^	-
	**Demethoxycurcumin (mg/g)**		
**Fresh**	40.27 ± 3.40 ^ab^	40.27 ± 3.10 ^ab#^	40.27 ± 3.40 ^a#^
**1 min**	31.63 ± 3.52 ^a^	35.33 ± 3.95 ^ab^	41.37 ± 4.48 ^a^
**2 min**	35.93 ± 4.61 ^ab^	45.30 ± 4.28 ^bc^	29.87 ± 3.80 ^a^
**5 min**	47.67 ± 4.44 ^bc^*^^^	28.50 ± 2.17 ^a^*^#^	11.96 ± 1.90 ^b^#^
**10 min**	56.87 ± 5.97 ^c^	55.13 ± 4.70 ^c^	-
	**Bisdemethoxycurcumin (mg/g)**		
**Fresh**	42.90 ± 3.87 ^a^	42.90 ± 3.87 ^ab^	42.90 ± 3.87 ^ab^
**1 min**	50.07 ± 4.20 ^a^	38.50 ± 4.88 ^a^	49.13 ± 5.30 ^b^
**2 min**	44.70 ± 3.05 ^a^	51.93 ± 4.01 ^b#^	32.93 ± 2.87 ^a#^
**5 min**	57.37 ± 5.50 ^a^*^^^	34.90 ± 3.31 ^a^*^#^	16.47 ± 1.96 ^c^#^
**10 min**	82.03 ± 7.00 ^b^	67.77 ± 5.03 ^c^	-

The means not sharing the same letter in a column are significantly different at *p* ≤ 0.05. Symbol explanations: *—the statistically significant difference between boiling and frying at *p* ≤ 0.05; ^—the statistically significant difference between boiling and microwaving at *p* ≤ 0.05; #—the statistically significant difference between frying and microwaving at *p* ≤ 0.05.

**Table 2:** Pearson’s correlation coefficient calculated for particular curcuminoids in the thermally processed turmeric rhizome.

Thermal Process	Bisdemethoxycurcumin	Demethoxycurcumin	Curcumin
**Boiling**	0.96	0.98	0.94
**Frying**	0.69	0.57	−0.83
**Microwaving**	−0.96	−0.99	−0.83

**Table 3:** Antioxidant activity of thermally processed turmeric rhizome.

	Boiling (*n* = 3)	Frying (*n* = 3)	Microwave (*n* = 3)
	TPC (mg/L)		
**Fresh**	410.00 ± 12.17 ^a^	410.00 ± 12.17 ^ab^	410.00 ± 12.17 ^a^
**1 min**	402.67 ± 68.39 ^a^	404.00 ± 44.19 ^ab^	491.00 ± 93.04 ^a^
**2 min**	419.00 ± 24.76 ^a^	468.00 ± 67.62 ^ab^	553.00 ± 79.08 ^a^
**5 min**	439.33 ± 65.25 ^a^	348.33 ± 74.39 ^a^	327.00 ± 117.22 ^a^
**10 min**	527.00 ± 34.39 ^a^	592.00 ± 88.27 ^b^	-
	**DPPH (%)**		
**Fresh**	39.65 ± 2.13 ^a^	39.65 ± 2.13 ^bc^	39.65 ± 2.13 ^a^
**1 min**	40.17 ± 3.12 ^a^	32.57 ± 1.90 ^ab#^	46.07 ± 3.89 ^a#^
**2 min**	42.13 ± 2.17 ^ab^	37.87 ± 5.53 ^abc^	47.87 ± 6.05 ^a^
**5 min**	43.43 ± 3.21 ^ab^*^^^	28.70 ± 2.97 ^a^*	19.73 ± 2.12 ^b^^
**10 min**	51.60 ± 4.00 ^b^	43.87 ± 4.81 ^c^	-

The means not sharing the same letter in a column are significantly different at *p* ≤ 0.05. Symbol explanations: *—the statistically significant difference between boiling and frying at *p* ≤ 0.05; ^—the statistically significant difference between boiling and microwaving at *p* ≤ 0.05; #—the statistically significant difference between frying and microwaving at *p* ≤ 0.05.

## Data Availability

Data is contained within the article or supplementary material.

## Supplementary Materials

The following supporting information can be downloaded at: https://www.mdpi.com/article/10.3390/foods12163086/s1, Figure S1: The obtained mass chromatogram of the total methanolic extract from turmeric rhizomes recorded in the negative ion mode with three curcuminoids eluted after 25.5 min together with their collected MS data; Figure S2: MS/MS spectra of three curcuminoids present in the analyzed extracts, in the negative ion mode; Figure S3: HPLC chromatograms recorded for the analysed samples that undergone the thermal processing by frying, microwave heating and boiling. The elution order: bisdemethoxycurcumin (BDMCC), demethoxycurcumin (DMCC), and curcumin (CC). References [48,49] are cited in the supplementary materials.
