# Supplementary material for: Impact of Thermal Processing on the Composition of Curcuma longa Rhizome
Source: Foods. 2023 Aug 17;12(16):3086. doi: 10.3390/foods12163086 (PMC10453412; doi:10.3390/foods12163086)
Supplement: Supplementary file 1 [file foods-12-03086-s001.zip › foods-2498688-supplementary.pdf]

SUPPLEMENTARY FILE

Thermal processing of turmeric and its impact on the composition of the rhizome material

Justyna Zagórska [1], Wirginia Kukula-Koch [2], Marcin Czop [3], Katarzyna Iłowiecka [1] and Wojciech Koch [1,*]

1 Department of Food and Nutrition, Medical University of Lublin, 4a Chodzki Str., 20-093 Lublin, Po-land; justyna.zagorska@umlub.pl (J.Z.), katarzyna.ilowiecka@umlub.pl (K.I.)

2 Department of Pharmacognosy with Medical Plants Garden, Medical University of Lublin, 1 Chodzki Str., 20-093 Lublin, Poland; virginia.kukula@gmail.com (W.K.-K.);

3 Department of Clinical Genetics, Medical University of Lublin, 11 Radziwiłłowska Str., 20-080 Lublin, Poland; marcin.czop@umlub.pl (M.C.)

* Correspondence: kochw@interia.pl (W.K.)

1. Conditions of the LC-MS analysis

LC-MS analysis of the samples was performed using previously described protocol with slight modifications [1]. Platform from Agilent Technologies (Santa Clara, CA, USA) on the HPLC column: Zorbax Eclipse Plus RP-18 (150 mm x2.1 mm; dp = 3.5 μm). It was particularly important to perform the HPLC-MS analysis to be sure about the identity and the elution order of the three curcuminoids which are eluted from the chromatographic column with very close Rf values. The instrument was equipped in a degasser (G1322A), an autosampler (G1329B), a binary pump (G1312C), a photodiode array detector (G1315D) and a mass spectrometer (G6530B)(Agilent Technologies, Santa Clara, CA, USA). For the analysis of data, an Agilent MassHunter Workstation Software (version B.10.00, Agilent Technologies, Santa Clara, CA, USA) was used. The chromatographic analysis was accomplished at the thermostat temperature of 20 ° C, in the UV detection wavelengths of 210, 230, 254, 280, and 320 nm, the range of detections of 190-600 nm. The injection volume was set at 10 μl, the flow rate of 0.2 mL / min and the analysis run at 45 min. The following gradient elution program of mobile phase B (acetonitrile with 0.1% of formic acid) in 0.1 % of formic acid in water was proposed: : 0 min - 30%B, 20 min - 45%B, 20.1 min - 70%B, 30-34 min - 95%B, 35 min - 30%B. Post run duration was set at 10 min. The settings of the mass spectrometer used in the study were as follows: the gas and sheath gas temperatures: 275 and 325 ◦C, gas flows: 12 L/min, capillary voltage: 3000 V, nozzle voltage: 1000 V, fragmentor voltage: 120 V, collision energies of 10 and 20 V, skimmer voltage: 65 V, nebulizer pressure: 35 psig, the mass range of 100-1700 u, the number of precursors per cycle: 2.

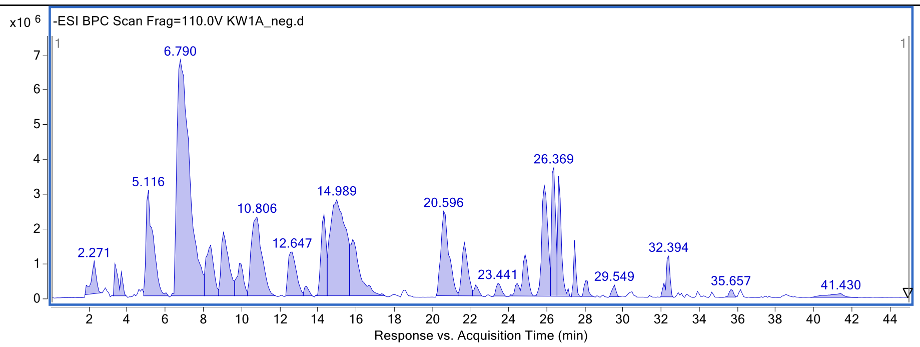

Ion (+/-)	Rt (min)	Molecular formula	m/z calculated	m/z experimental	Delta (mmu)	DBE	Proposed compound
$[M-H]^-$	25.7	$C_{19}H_{16}O_4$	307.0976	307.0973	-0.92	12	Bisdemethoxycurcumin
$[M-H]^-$	26.3	$C_{20}H_{18}O_5$	337.1087	337.1022	-0.44	12	Demethoxycurcumin
$[M-H]^-$	26.7	$C_{21}H_{20}O_6$	367.1187	367.1187	-0.03	12	Curcumin

Figure S1. The obtained mass chromatogram of the total methanolic extract from turmeric rhizomes recorded in the negative ion mode with three curcuminoids eluted from 25.5 min together with their collected MS data.

The three curcuminoids of interest, curcumin, demethoxycurcumin and bisdemethoxycurcumin were detected in the mass chromatograms after around 26th minutes. The following MS/MS spectra were obtaiend for the three compounds.

Curcumin

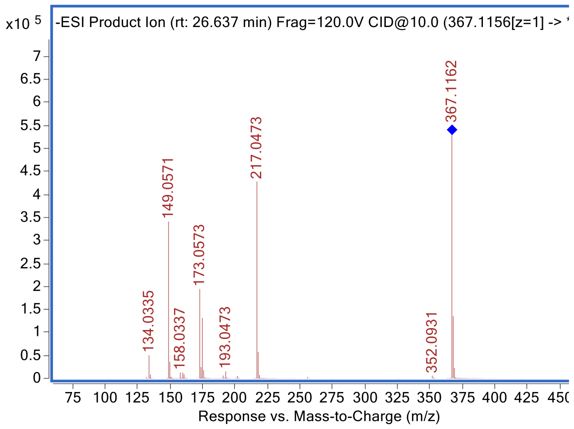

Demethoxycurcumin

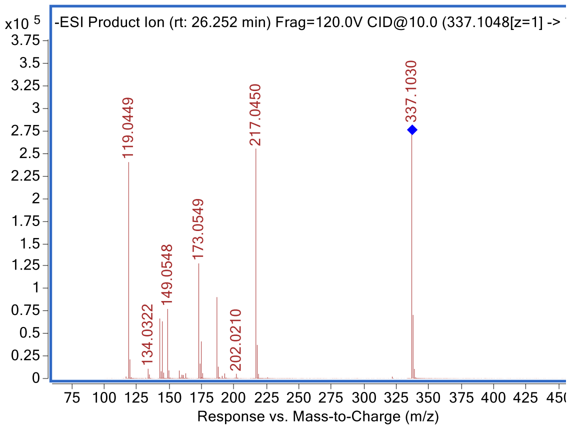

Bisdemethoxycurcumin

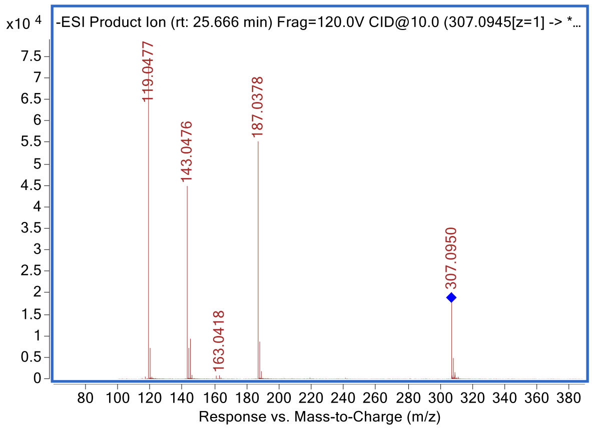

Figure S2. MS/MS spectra of three curcuminoids present in the analyzed extracts, in the negative ion mode.

The obtained MS/MS spectra resembled those described by other authors [2]. For bisdemethoxycurcumin and demethoxycurcumin the m/z signal of 119 was visible, that was coming from the dettachment of one cinnamic group. The MS/MS spectrum of curcumin, that is characterized by the presence of methoxyl-substituted group at both terminal rings showed the *m/z* of 149 that was characteristic for a fragment with removed one methoxylated cinnamic group. Curcumin and demethoxycurcumin spectra showed the presence of a 217 *m/z* signal, whereas the spectra of bisdemethoxycurcumin contained the *m/z* of 187 that stayed after the removal of 119 fragment.

High accuracy mass measurement, composition of MS/MS spectra, measured double bond equivalent numbers and the low mass error value provided sufficient data for the identification of the three curcuminoids. The elution order of the three curcuminoids on the RP-18 chromatographic column was: bisdemethoxycurcumin, demethoxycurcumin and curcumin.

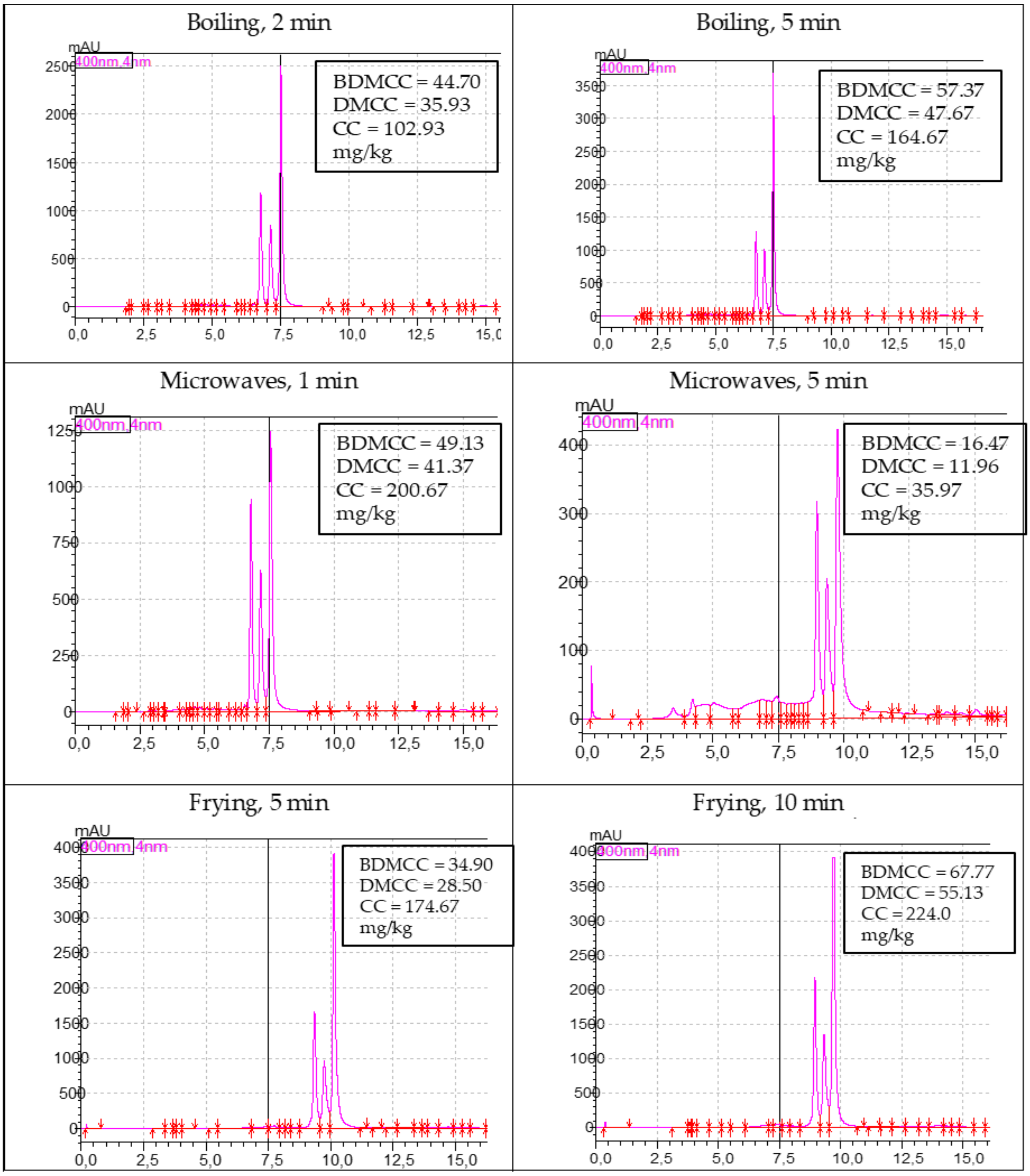

Figure S3. HPLC chromatograms recorded for the analysed samples that undergone the thermal processing by cooking, microwave heating and boiling. The elution order: bisdemethoxycurcumin (BDMCC), demethoxycurcumin (DMCC), and curcumin (CC).

References

1. Kukula-Koch, W.; Grabarska, A.; Łuszczki, J.; Czernicka, L.; Nowosadzka, E.; Gumbarewicz, E.; Jarząb, A.; Audo, G.; Upadhyay, S.; Głowniak, K.; Stepulak, A. Superior anticancer activity is demonstrated by total extract of

Curcuma longa L. as opposed to individual curcuminoids separated by centrifugal partition chromatography. Phytother. Res. **2018**, 32(5), 933-942. doi: 10.1002/ptr.6035.

2. Liu, Y.; Siard, M.; Adams, A.; Keowen, M.L.; Miller, T.K.; Garza, F.; Andrews, F.M.; Seeram, N.P. Simultaneous Quantification of Free Curcuminoids and Their Metabolites in Equine Plasma by LC-ESI-MS/MS. *J Pharm Biomed Anal* **2018**, *154*, 31–39, doi:10.1016/j.jpba.2018.03.014.
